# Assessing the Effects of Vitamin D on Neural Network Function in Patients With Parkinson’s Disease by Measuring the Fraction Amplitude of Low-Frequency Fluctuation

**DOI:** 10.3389/fnagi.2021.763947

**Published:** 2021-12-20

**Authors:** Lingling Lv, Hainan Zhang, Xuling Tan, Lixia Qin, Xinke Peng, Rongrong Bai, Qile Xiao, Changlian Tan, Haiyan Liao, Weiqian Yan, Jieqiong Tan, Beisha Tang, Chunyu Wang

**Affiliations:** ^1^Department of Neurology, The Second Xiangya Hospital, Central South University, Changsha, China; ^2^Department of Medical Genetics, The Second Xiangya Hospital, Central South University, Changsha, China; ^3^Department of Radiology, The Second Xiangya Hospital, Central South University, Changsha, China; ^4^Center for Medical Genetics, School of Life Sciences, Central South University, Changsha, China; ^5^Hunan Key Laboratory of Animal Models for Human Diseases, Central South University, Changsha, China; ^6^Hunan Key Laboratory of Medical Genetics, Central South University, Changsha, China; ^7^National Clinical Research Center for Geriatric Disorders, Xiangya Hospital, Changsha, China; ^8^Department of Neurology, Xiangya Hospital, Central South University, Changsha, China; ^9^Key Laboratory of Hunan Province in Neurodegenerative Disorders, Central South University, Changsha, China

**Keywords:** Parkinson’s disease, vitamin D, risk, resting-state functional MRI, fraction amplitude of low-frequency fluctuation

## Abstract

**Background:** Recently, many studies have shown that low vitamin D (VD) levels may be related to an increased risk of Parkinson’s disease (PD), but the underlying mechanisms remain unclear.

**Objective:** To explore the relationship between PD and VD levels, as well as to analyze the effects of VD on spontaneous brain activity and explore the possible mechanism of its involvement in PD risk.

**Methods:** In a cross-sectional study, we quantified the difference in VD levels between 330 PD patients and 209 healthy controls (HC) to explore the correlation between VD and PD risk. We also acquired resting-state Functional Magnetic Resonance Imaging (rs-fMRI) data from 46 PD patients and 21 HC. The PD patients were divided into three groups according to 25(OH)D levels: PD patients with VD deficiency (PD + VDD), PD patients with VD insufficiency (PD + VDI), and PD patients with normal VD (PD + NVD). The effect of VD status on spontaneous neuronal activity in the whole brain was analyzed by measuring the fraction amplitude of low-frequency fluctuation (fALFF).

**Results:** Compared with HC, the PD patients had lower serum 25(OH)D levels (23.60 ± 7.27 vs. 25.60 ± 5.78, *P* < 0.001). The 25(OH)D level may have a potential dose-dependent effect on the risk of PD (*P*_*trend*_ = 0.007). A high risk of PD was associated with VD deficiency [25(OH)D < 20 ng/mL, OR = 2.319], and the lowest quartile of 25(OH)D concentration was associated with a high risk of PD (OR = 1.941). In the rs-fMRI study, PD + VDD patients had wider brain regions with altered fALFF than other PD groups when compared with the corresponding HC groups. Both PD + VDD and PD + VDI showed higher fALFF in the cuneus, left precuneus, calcarine cortex and right lingual, as well as lower fALFF in the left middle temporal gyrus. PD + VDD patients also showed higher fALFF in the left superior, middle and inferior frontal gyri, as well as the left precentral gyrus than HC. Among PD patients, there was only a statistically significant difference in fALFF between the PD + VDD and PD + NVD groups. Compared with the PD + NVD group, PD + VDD patients exhibited higher fALFF in the left precentral and left postcentral gyrus, as well as the left inferior parietal lobule.

**Conclusion:** These results demonstrate that PD patients had lower serum VD levels than HC, and VD may have a potential dose-dependent effect on PD risk. Lower serum VD levels can affect the spontaneous neuronal activity of default-mode network (DMN) and visual pathway neurons in PD patients, providing a possible mechanism for its effect on PD risk.

## Introduction

Parkinson’s disease (PD) is a common neurodegenerative disorder associated with the loss of dopaminergic neurons in the substantia nigra and the formation of Lewy bodies within neurons (Ascherio and Schwarzschild, 2016; Armstrong and Okun, 2020). However, the exact cause of this crucial pathological feature is still unclear. The pathogenesis likely involves aging, environmental factors and genetic factors (Logroscino, 2005). Among them, environmental factors, especially vitamin D (VD), are thought to be related to the occurrence of PD (Shen and Ji, 2015; Wang et al., 2016). Accumulating evidence from *in vivo* and *in vitro* animal studies suggest that VD plays a neuroprotective and nutritional role in dopaminergic neurons by regulating neurotrophic factors, inducing the removal of α-synuclein, maintaining calcium homeostasis, as well as regulating oxidative stress and the immune response (Aureli et al., 2014; Calvello et al., 2017; Phillipson, 2017; Pertile et al., 2018).

Studies have found that VD deficiency [25(OH)D < 20 ng/mL] is more common among PD patients than healthy controls (HC) (Suzuki et al., 2012; Ding et al., 2013). Reduced mobility and gastrointestinal dysfunction may be related to the higher incidence of VD deficiency in PD patients (Liu and Zhang, 2013), but some studies have found that the incidence of VD insufficient in early PD patients with normal gastrointestinal function and unrestricted activity is also significantly higher than in HC (Evatt et al., 2011). Therefore, VD deficiency may not simply be a consequence of PD, but may also be related to the occurrence and development of the disease (Newmark and Newmark, 2007; Peterson, 2014). There is increasing evidence that VD deficiency has a long latency or a slow effect, which may be important for late-onset PD. A 29-year prospective study in Finland confirmed that low serum 25(OH) D levels are related to a high risk of PD (Knekt et al., 2010; Shen and Ji, 2015; Wang et al., 2016), but this was not confirmed in all studies (Shrestha et al., 2016; Larsson et al., 2017), and the mechanism is not clear. Therefore, further studies are warranted in this area to explore the relationship between VD and PD risk. As changes in the brain’s structure and function caused by VD deficiency can be detected using rs-fMRI, we investigated the impact of VD on neural network function to explore the possible mechanism of its involvement in PD risk. The findings of this study provide important theoretical insights for the prevention of PD.

## Materials and Methods

### Study Subjects

The first cohort, the case-control sample, comprised 330 idiopathic PD patients and 209 HC who visited the Second Xiangya Hospital between July 2018 and December 2020. The second cohort, the PD-MRI cohort, randomly selected from the first cohort, comprised 46 individuals with PD and 21 HC who took part in the study and underwent MRI scans. The idiopathic PD patients were diagnosed strictly according to the Movement Disorder Society (MDS) Clinical Diagnostic Criteria for PD (Postuma et al., 2015). PD patients with a course of disease less than 3 years at the time of enrollment were followed up for at least 12 months to ensure their course of the disease lasts more than 3 years at the end of the study. And the diagnosis was reassessed and confirmed at the end of follow-up. At enrollment, we excluded patients with the following features: participants with secondary Parkinson-plus syndromes; Parkinsonism; genetically predisposed PD; use of VD supplements; history of any-type of chronic disease and/or use of drugs with a potential impact on the absorption of VD; patients with incomplete data. At the same time, the sex- and age-matched HC were recruited from the community. All controls were cognitively normal, with no PD symptoms and no history of neurological disease. A specific exclusion criterion for the HC group was that a first- or second-degree relative had PD, and the remaining exclusion criteria were the same as in the case group. The Ethics Committee of The Second Xiangya Hospital of Central South University approved the cross-sectional study and all the subjects provided their informed written consent to participate.

### Clinical Assessments

The demographic characteristics and clinical features of HC and PD patients, including age, age at onset, sex, years of education, height and weight were recorded. The disease duration and drug history of PD patients were recorded in detail, and after which the levodopa equivalent daily dose (LEDD) was calculated. The disease severity and cognitive functional status of PD patients were evaluated in the “OFF” condition through in-person interviews using the Unified Parkinson’s Disease Rating Scale part III (UPDRS-III), the Hoehn and Yahr Staging Scale (H&Y), and the Mini-Mental State Examination (MMSE).

Blood samples and the resting-state functional magnetic resonance imaging (rs-fMRI) data were acquired on the same day as the neuropsychological testing was conducted. Serum 25(OH)D levels were measured using a liquid chromatography tandem mass spectrometry (LC-MS/MS) method, which reported 25(OH)D standardized results (Bivona et al., 2019c). The testing process included: (1) preparation of alternative calibration matrix (4% Bovine Serum Albumin, 4% BSA); (2) preparation of calibration solution (25-hydroxyvitamin D_3_ and 25-hydroxyvitamin D_2_ from the company of Sigma-Aldrich, the purity ≥ 98.0%) and isotope internal standard solution ([26,26,26,27,27,27-d6]-25(OH)D_2_, d6-25(OH)D_2_, purity ≥ 99.0%) and ([26,26,26,27,27,27-d6]-25(OH)D_3_, d6-25(OH)D_3_, purity ≥ 99.0%) from the company of Medical Isotopes); (3) drawing a standard curve [A certain volume of 25 (OH) D_2_ and 25 (OH) D_3_ standard substance solutions were added into the 4% BSA to prepare standard solutions with concentrations of 2.20, 3.13, 6.25, 12.50, 25.00, 60.00, and 100.00 ng/mL, respectively, and the standard curve was drawn]; (4) sample processing and detection: liquid–liquid extraction was used to extract the measured substance from the serum to the maximum extent, and then further purification and separation by liquid chromatography, and finally qualitative and quantitative by tandem mass spectrometry. Based on established clinical criteria (Holick et al., 2011), the PD patients were grouped according to their 25(OH)D levels into: PD patients with VD deficiency (25[OH]D < 20 ng/mL, PD + VDD), PD patients with VD insufficiency (20 ng/mL < 25[OH]D < 30 ng/mL, PD + VDI), and PD patients with normal VD levels (25[OH]D > 30 ng/mL, PD + NVD).

### MRI Study

The rs-fMRI data were recorded using a MAGNETOM Skyra 3T MRI (Siemens, Germany). The Echo Planar Imaging (EPI) sequence (TR = 2500 ms, TE = 25 ms, FA = 90°, matrix = 64 × 64, FOV = 240 mm × 240 mm, slice thickness = 3.5 mm, slices = 39, no slice gap in the resting condition), was used to record the blood oxygenation level-dependent (BOLD) signal. The Axial T1 structural images were acquired using the T1WI three-dimensional magnetization prepared rapid acquisition gradient echo (T1WI 3D MP-RAGE) sequences (repetition time [TR] = 1,900 ms, echo time [TE] = 2.01 ms, flip angle [FA] = 9°, slice thickness = 1 mm, slices = 176, field of view [FOV] = 256 mm × 256 mm, matrix size = 256 × 256). During the MRI scan, the subject’s eyes were closed, but avoided falling asleep, and the head was stabilized with foam pads to reduce noise. Rs-fMRI scans were conducted in the ‘‘OFF’’ condition after neuropsychological assessments. DPABI and REST software based on MATLAB R2019B was used to preprocess the MRI data^1^ (Yan et al., 2016). The single subject pre-processing included: convert data from DICOM format to NIFTI format, removal of the first 10 time points, time and space correction, head motion correction, registration, segmentation, transformation and normalization of the resulting aligned data to the Montreal Neurological Institute (MNI) space with the segmented information, followed by resampling to a voxel size of 3 mm × 3 mm × 3 mm, spatial smoothing with an isotropic 6-mm full width at half maximum (FWHM) Gaussian kernel. After normalization and smoothing, the waveform of each voxel was finally used for remove the linear trends of time courses and for temporal band-pass filtering (0.01–0.08 Hz). Patients with the translation of more than 1.5 mm and a rotation angle of more than 1.5° during the course of the scan were excluded.

### Statistical Analysis

Statistical analyses were performed in SPSS version 25.0 for macOS (IBM Corp., United States). Continuous variables conforming to the normal distribution and homogeneity of variance were compared between two groups using Student’s *t-*test, or analysis of variance (ANOVA) for comparisons between three or more groups. Otherwise, the Mann–Whitney *U* test and Kruskal–Wallis test were used, respectively. The χ^2^ statistic or Fisher’s exact test were used to evaluate the differences of categorical variables. For the correlation analyses, multiple regression analysis controlled for age and gender was used to identify the MMSE associated with VD in PD. Logistic regression analysis was used to evaluate the association between VD concentrations and the risk of PD, adjusted for age at sample collection, BMI, sex, and sampling season. Differences with *P*-values < 0.05 were considered statistically significant, and all statistical tests were two-tailed. The strength of association was interpreted using odds ratios (OR), and we also calculated prevalence with 95% confidence intervals.

For rs-fMRI data analysis, the intergroup differences were compared using the voxel-wise two-sample *t*-test embedded in SPM software (Yan et al., 2016) with gender and age as covariates. During fraction amplitude of low-frequency fluctuation (fALFF) analysis, the GRF method was used to perform multiple comparison corrections between PD subgroups (PD + VDD, PD + VDI, and PD + NVD) and the HC group (*P* < 0.01). The AlphaSim method was used to perform multiple comparison corrections between PD subgroups, *P*-value < 0.05 after correction.

## Results

### Clinical Characteristics and 25(OH)D Levels of Different Groups

The detailed demographic characteristics of the two groups are shown in Table 1. Among the PD cases 271 (82.1%) patients were classified into early PD (H&Y stage 1–2.5) and 59 (17.8%) into mid- and late PD (H&Y stage 3–5). In our dataset, there was no significant difference in terms of age, sex, BMI and sampling season between cases and controls.

**TABLE 1:** The demographic and clinical data of Parkinson’s disease patients and health controls (HC).

Variable	PD (*N* = 330)	HC (*N* = 209)	*P*-value
Gender: M/F	180/150	127/82	0.155^b^
Age (years)	59.09 ± 9.97	60.72 ± 13.00	0.102^c^
Onset age (years)	55.86 ± 10.08	NA	NA
BMI (kg/m^2^)	22.83 ± 3.12	22.77 ± 3.00	0.843^c^
Educational level (years)	8.08 ± 4.47	NA	NA
Disease duration (months)	24 (12, 48)	NA	NA
H&Y stage^a^	271/59	NA	NA
UPDRS-III score	19 (11, 28.25)	NA	NA
MMSE	24.23 ± 3.84	NA	NA
Sampling season (July–December; %)	239 (72.4%)	159 (76.1%)	0.347^b^

*^a^Stages 1–2.5/stages 3–5, cases; ^b^χ^2^ test; ^c^two student’s T test; PD, Parkinson’s disease; HC, health controls; BMI, body mass index; MMSE, Mini-Mental status examination; NA, not applicable.*

**FIGURE 1:**
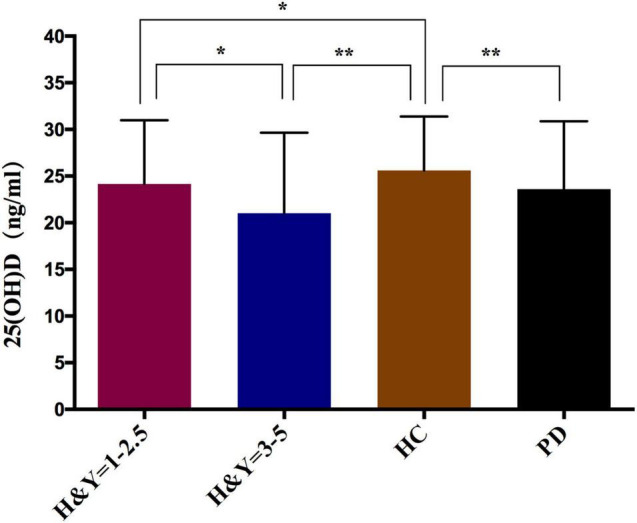
Comparison of serum 25(OH)D levels in PD patients and healthy controls. *p < 0.05, **p < 0.01; *P*-values were calculated using Student‘s *t*-test.

Our results showed that the serum 25(OH)D levels of PD patients were lower than in the HC group (23.60 ± 7.27 vs. 25.60 ± 5.78, *P* < 0.001) (Table 2). Next, the severity of PD was evaluated according to the H&Y stage (1–5), and when the PD patients were divided into early (H&Y stage = 1–2.5) and middle or late PD (H&Y stage = 3–5), there was a significant trend showing that as the HY stage worsened, the 25(OH)D levels became lower. In H&Y stages 1–2.5, the 25(OH)D level was 24.16 ± 6.83, compared to 21.03 ± 8.61 for H&Y stages 3–5 (*P* = 0.011). Moreover, advanced PD patients had significantly lower 25(OH)D levels than HC (21.03 ± 8.61 vs. 25.60 ± 5.78, *P* < 0.001). The early PD patients had similar results (24.16 ± 6.83 vs. 25.60 ± 5.78, *P* = 0.043) (Figure 1). Interestingly, we also found that the 25(OH)D concentration was significantly higher in male PD patients than in female patients (*P* = 0.006), while there was no gender difference in VD levels among the HC (Table 2).

**TABLE 2:** Serum 25(OH)D levels in different populations.

	25(OH)D (ng/ml)
PD	23.60 ± 7.27*
HC	25.60 ± 5.78
H&Y stages: 1–2.5	24.16 ± 6.83*^#^
H&Y stage: 3–5	21.03 ± 8.61*
PD: male	24.60 ± 7.30**
PD: female	22.40 ± 7.08
HC: male	25.73 ± 5.75
HC: female	25.41 ± 5.86

P-values are calculated using two-tailed Student’s t-test; *compared to HC (p < 0.05); ^#^Compared to H&Y stage: 3–5 (p < 0.05); **compared with the female PD patients (p < 0.05).

### Vitamin D Level and Parkinson’s Disease Risk

The 25(OH)D level may have a potential dose-dependent effect on the risk of PD (*P*_*trend*_ = 0.007) (Table 3). There was no significant correlation between VD insufficiency and the risk of PD, but VD deficiency was clearly associated with the risk of PD. Individuals with PD were 2.319 times more likely to be VD deficient than controls (95% CI, 1.501–3.583). Furthermore, the lowest quartile of 25(OH)D concentration was significantly associated with a high risk of PD relative to the highest quartile (OR = 1.941; 95% CI, 1.140–3.305).

**TABLE 3:** Association between vitamin D (VD) and PD risk.

	OR	95% CI	*P*-value
Vit D deficiency [25(OH)D < 20 ng/ml]	2.319	(1.501, 3.583)	0.000
Vit D insufficiency [25(OH)D < 30 ng/ml]	0.964	(0.624, 1.490)	0.869
25(OH)D			0.007 (*P*_*trend*_)^a^
Q1 (6.2–19.9 ng/ml)	1.941	(1.140, 3.305)	0.015
Q2 (19.9–24.8 ng/ml)	0.800	(0.490, 1.305)	0.371
Q3 (24.8–28.5 ng/ml)	0.750	(0.452, 1.243)	0.264
Q4 (>28.5 ng/ml)	Reference		

*Logistic regression analysis was used to evaluate the association between serum VD levels and the risk of PD, adjusting for age at sampling, BMI, sex, and sampling season; ^a^χ^2^ trend test.*

### Resting-State Functional Magnetic Resonance Imaging Results

**FIGURE 2:**
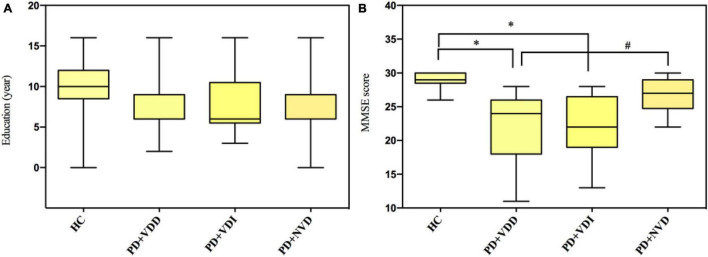
Comparison amounts of the PD + VDD, PD + VDD, and PD + NVD patients and the health control group with Bonferroni correction; **(A)** comparison of education in PD patients and healthy controls; **(B)** comparison of MMSE scores in PD patients and healthy controls. ^#^*P* = 0.029, *P < 0.001; PD + VDD, PD patients with VD deficiency; PD + VDI, D patients with VD insufficiency; PD + NVD, PD patients with normal VD levels; MMSE, Mini-Mental State Examination.

Next, we evaluated the effects of VD levels on resting state brain function using rs-fMRI scans. The demographic and clinical characteristics of the PD subgroups and HC group are shown in Table 4 and Figure 2. There were no significant differences in age and sex between the HC group and PD subgroups (*p* > 0.05). Furthermore, no statistically significant differences were observed among the PD subgroups for disease duration, UPDRS-III scores and H&Y stage (*p* > 0.05). In terms of MMSE score, although no statistically significant differences were observed between the two groups for years of education (Figure 2A), there was a statistically difference between the PD + NVD and PD + VDD groups [27(25, 29) vs. 24(18, 26), *P* = 0.029] (Figure 2B).

**TABLE 4:** Demographic characteristics of the PD patients and health controls.

	HC (*N* = 21)	PD (*N* = 46)			*P*-value
		PD + VDD (*n* = 19)	PD + VDI (*n* = 13)	PD + NVD (*n* = 14)	
Sex (male/female)	12/9	10/9	6/7	12/2	0.146
Age (year)^a^	56 (52, 61)	54 (49, 63)	61 (57, 66)	56 (50, 66)	0.367
Education (year)^a^	10 (9, 12)	6 (6, 9)	6 (6, 9)	9 (6, 9)	0.030
Disease duration (month)^a^	NA	24 (12, 60)	12 (5, 36)	24 (12, 48)	0.445
UPDRS-3 score^a^	NA	18 (11, 38)	16 (11, 20)	26 (14, 43)	0.306
H&Y score^a^	NA	1.5 (1, 2.5)	2 (1.5, 2)	2.25 (1.5, 3)	0.405
MMSE score^a^	29(29, 30)	24 (18, 26)	22 (19, 26)	27 (25, 29)	0.000

*^a^Median (interquartile range). Sex data were compared using Pearson’s chi-squared. Age, Education, Disease duration, UPDRS-3, H&Y score and MMSE score data were compared using the Kruskal–Wallis test. PD + VDD, PD patients with VD deficiency; PD + VDI, PD patients with VD insufficiency; PD + NVD, PD patients with normal VD levels; MMSE, Mini-Mental status examination.*

**FIGURE 3:**
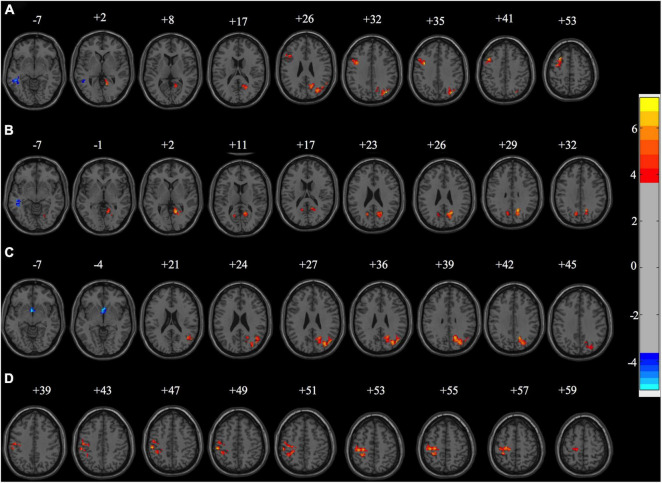
Fraction amplitude of low-frequency fluctuation differences between the sub-groups of PD patients and healthy controls (HC). **(A)** fALFF differences between PD + VDD and HC (corrected for multiple comparisons, at GRF, *p* < 0.01). **(B)** fALFF differences between PD + VDI and HC (corrected for multiple comparisons, at GRF, *p* < 0.01). **(C)** fALFF differences between PD + NVD and HC (corrected for multiple comparisons, at GRF, *p* < 0.01). **(D)** fALFF differences between PD + VDD and PD + NVD (corrected for multiple comparisons, at AlphaSim, *p* < 0.05). Red: increased fALFF. Blue: decreased fALFF.

In the rs-fMRI study, we first compared the fALFF map of PD subgroups to controls. We found that there were significant differences between the PD subgroups and HC in fALFF power (Figure 3). Compared with the HC group, the PD + VDD group exhibited increased fALFF power in the left superior, middle and inferior frontal gyrus, right angular gyrus, left precentral gyrus, right precuneus, right cuneus, right lingual gyrus, right calcarine fissure cortex, as well as the right superior and middle occipital gyrus. These regions largely overlapped with the so-called default-mode network (DMN). In addition, there was decreased fALFF power in the right middle temporal gyrus (corrected for multiple comparisons, at GRF, *p* < 0.01, Figure 3A). We then compared the fALFF maps of PD-VDI and HC, and found that some regions (calcarine fissure cortex, precuneus, cuneus, and right lingual gyrus) exhibited increased fALFF power, while other parts exhibited a decrease (left, middle and inferior temporal gyrus) (corrected for multiple comparisons, at GRF, *p* < 0.01, Figure 3B). We also compared PD + NVD to controls, and found that some brain areas showed increased fALFF power in PD + NVD, including the right precuneus, right cuneus, right middle temporal gyrus, right angular gyrus, as well as the right superior and middle occipital gyrus. Areas showing decreased fALFF in the PD + NVD group included the nucleus accumbens (ventral striatum) and the left olfactory cortex (corrected for multiple comparisons, at GRF, *p* < 0.01, Figure 3C). Among the PD subgroups, only the difference between the PD + VDD and PD + NVD groups was statistically significant (AlphaSim correction, *P* < 0.05). Compared with the PD + NVD group, the brain regions with increased fALFF power in the PD + VDD group included the left precentral gyrus, left postcentral gyrus, and left inferior parietal lobule (left superior marginal gyrus and left angular gyrus). No areas of the brain with reduced fALFF were observed (Figure 3D).

## Discussion

The current study investigated the relationship between VD, PD risk, and neural network function in individuals with PD through a combination of rs-fMRI and quantitative analysis of serum 25(OH)D levels. We found that serum VD levels were lower in PD patients and were inversely associated with the PD risk. This was in agreement with several previous studies (Knekt et al., 2010; Zhu et al., 2014; Wang et al., 2015), but the exact mechanism is unclear. To our best knowledge, this is the first case-control study designed to evaluate changes of fALFF related to VD, and to tentatively determine the mechanism by which VD influences the risk of PD. The present rs-fMRI study demonstrated that the BOLD signal is altered in PD patients and that these fALFF alterations in DMN and visual pathway neurons are associated with the serum levels of VD. These associations may reflect a possible key role of VD in the development of PD.

The present study found that circulating 25(OH)D levels were lower in PD patients than HC (23.60 ± 7.27 vs. 25.60 ± 5.78). This result was in agreement with previous studies (Evatt et al., 2008; Suzuki et al., 2012; Liu and Zhang, 2013), but was not confirmed in all studies. A study conducted in the Faroe Islands showed that although 25(OH)D levels in PD patients were slightly lower than in the HC group, the difference was not statistically significant (Petersen et al., 2014). It is likely that geographical factors and dietary habits of the Faroe Islands contribute to the prevalence of low VD levels in the population. The present study also found that the serum levels of 25(OH)D were higher in male than in female patients, which may be related to the fact that male patients are engaged in more outdoor work. Previous studies have also shown that outdoor work and increased sunlight exposure can increase serum 25(OH)D levels in PD patients (Zhu et al., 2014; Wang et al., 2016). This can be explained by the fact that 80% of serum VD is synthesized in the skin via a mechanisms that depends on solar ultraviolet B radiation (Wang et al., 2016).

In agreement with the literature (Suzuki et al., 2012), our findings indicate that lower 25(OH)D levels were significantly associated with higher H&Y stages. The immobility and gastrointestinal dysfunction of PD patients becomes more severe as the disease progresses, which likely results in decreased synthesis and uptake of VD in the body. Hence, the long-term effects of PD could lead to a decrease in VD levels. However, low levels of VD can in turn affect PD patients, especially those with advanced PD, since VD deficiency is an important factor in the development of fractures and osteoporosis (Park et al., 2019). A previous study found that daily supplementation with 700–1,000 IU of VD reduced the incidence of falls by about 19% (Bischoff-Ferrari et al., 2009). Therefore, PD patients should be included in a high-risk population with VD deficiency, and be tested for VD levels and given early intervention.

The present study also found that the levels of 25(OH)D in patients with early PD were also lower than in the HC group (24.16 ± 6.83 vs. 25.60 ± 5.78, *P* < 0.05). Consistently, Evatt et al. (2011) found that early PD patients with normal gastrointestinal function and unrestricted activity also had a higher prevalence of VD deficiency than the general population. However, the level of VD did not decline further during the follow-up period, but increased slightly, suggesting that the level of VD may not decrease further with disease progression in the early stage of PD (Evatt et al., 2011). Notably, the limited mobility and gastrointestinal dysfunction common in advanced PD cannot explain the lower VD levels in patients with early PD. In addition, VD plays a neuroprotective and nutritional role in dopaminergic neurons. Therefore, it is hypothesized that VD deficiency may not only be a consequence of PD, but may also be a factor in its occurrence and progression (Newmark and Newmark, 2007).

The present study extends and refines the hypothesis that low serum VD could be a risk factor for PD, which was in line with previous studies (Knekt et al., 2010; Zhu et al., 2014; Wang et al., 2015). We found that a high risk of PD was associated with VD deficiency (25[OH]D < 20 ng/ml) (OR = 2.319, *P* < 0.001), but not with VD insufficiency (25[OH]D < 30 ng/ml), which suggests that the effect of VD on the risk of PD may be related to its concentration. We further found that the prevalence of PD in the lowest quartile of 25(OH)D concentrations was twice as high as in the highest quartile. Moreover, a whole-brain voxel-wise analysis of PD subgroups indicated that there were no brain regions that showed significant between-group differences except for the fALFF difference between the PD + VDD and PD + NVD groups. This further supports the possibility of a dose-dependent effect of VD on the risk of PD. We therefore speculated that VD may influence PD risk by affecting the spontaneous activities of specific brain regions. However, not all studies agree with our findings. For example, a large-sample case-control study found that VD insufficiency was also significantly associated with the risk of PD (OR = 2.13) (Wang et al., 2015). Furthermore, studies suggested that VD serum levels could not predict the risk of PD (Shrestha et al., 2016; Larsson et al., 2017). We therefore believe that this difference may be due to differences in the HC group and the size of the sample between studies. Moreover, it should also be noted that differences in VD analytical methods and lack of standardized 25(OH)D data can also lead to high heterogeneity among these results (Bivona et al., 2019c). Therefore, more prospective cohort studies with larger sample sizes are needed to clarify the role of serum VD levels in the occurrence and progression of PD. Importantly, further studies should use internationally recognized measurement procedures and materials to measure VD levels.

Several studies (Knekt et al., 2010; Zhu et al., 2014; Wang et al., 2015), including ours, indicated that serum VD levels are related to the risk of PD, but the exact mechanisms mediating this link are poorly understood. Substantial advances in the understanding of the PD pathology indicate that it likely involves a series of events including oxidative stress, misfolded proteins, mitochondrial dysfunction, neuroinflammation, immune regulation, and pathological changes (Ahlskog, 2005; Ghavami et al., 2014; Kalia and Lang, 2015). Additionally, studies also indicate that VD may play key roles in antioxidant defenses, immunomodulation, homeostasis of calcium, zinc, iron and manganese, as well as synaptic plasticity and the physiology of the dopaminergic system in the nervous system (Groves et al., 2014; Lima et al., 2018; Bivona et al., 2019a,b; Mayne and Burne, 2019). Animal models suggest that treatment with 1,25(OH)_2_D_3_(active VD) can partially protect DA neurons against the effects of intraventricularly administered 6-hydroxydopamine (6-OHDA) (Smith et al., 2006). Accordingly, VD can exert neuroprotective effects, which might support the view that low VD levels may increase the risk of PD through direct or indirect pathophysiological mechanisms.

We found progressive underlying changes in spontaneous neuronal activity in the PD + NVD, PD + VDI, and PD + VDD groups. Lower VD levels have a greater impact on the spontaneous activity of neurons. More specifically, the regions showing increased fALFF under the influence of low VD levels include the left superior, middle and inferior frontal gyri, cuneus, left precuneus, calcarine cortex, right lingual gyrus, left precentral gyrus, right lingual gyrus and calcarine cortex, as well as the left inferior parietal lobule. Moreover, the patient groups with lower VD levels showed a predominantly decreased fALFF in the left middle and inferior temporal gyrus. This dose-dependent relationship between VD and fALFF indicates that the cerebral cortex may be vulnerable to VD deficiency, and highlights the value of VD levels in the evaluation of the neural network function in PD. In the absence of disease, there are extensive neural network connections between the cerebral cortex, striatum, and thalamus, while in PD patients, there is progressive loss of dopaminergic (DA) neurons in the substantia nigra (SN), leading to a decrease in dopamine transmission in the striatum and an abnormal cortical-striatum circuit. Converging evidence suggests that VD is an essential regulator of physiological processes in dopaminergic neural circuits. Many studies in mouse models of PD have found that treatment with 1,25(OH)_2_D_3_ resulted in an increase of vitamin D receptor (VDR) expression and tyrosine hydroxylase (TH), as well as increasing the dopamine content (Smith et al., 2006; Jiang et al., 2014; Li et al., 2015). Furthermore, the VDR is expressed widely in the striatum and cortex of the adult brain, and the metabolizing enzymes that support VD activity are also expressed in neurons and glial cells (Fernandes de Abreu et al., 2009). Taken together, VD plays a crucial role in the cortical-striatum circuit by influencing the dopamine production pathways.

There are several possible mechanisms by which VD effects PD. Firstly, under inflammatory stimulation or oxidative stress, 1,25(OH)_2_D_3_ interacts with the nuclear VDR, which can directly or indirectly upregulate and downregulate the expression of target genes in the brain (Cui et al., 2017), including glial-derived nerve growth factor (GDNF) (Pertile et al., 2018), TGF-β (Bivona et al., 2019a), SLC30A10 and SLC39A2 (Claro da Silva et al., 2016), L-type Ca^2+^ channels (L-VGCC) (Phillipson, 2017), and γ-glutamyl-transpeptidase (Garcion et al., 1999). Besides, VDR also has non-transcriptional effects in calcium-and kinase-activated signaling pathways (Cui et al., 2017). Additionally, 1,25(OH)_2_D_3_ can interact with membrane receptor-endoplasmic reticulum stress protein 57 (ERp57) to exert neuroprotective effects (Cui et al., 2017). In the PD model induced by 6-OHDA, the level of ERp57 protein in the striatum was increased (Aureli et al., 2014), which can prevent the misfolding and aggregation of α-synuclein, control the quality of protein processing, maintain Ca^2+^ homeostasis and regulate cellular stress responses (Grillo et al., 2006). Taken together, VD may play a significant role in the pathophysiology of PD by genetic and non-transcriptional routes. However, the accumulation of misfolded alpha-synuclein, a major hallmark of PD (Obeso et al., 2017), was not evaluated in the present study. Therefore, further validation studies should be conducted.

We also found that the DMN and visual pathway neurons showed increased fALFF, while a part of the temporal lobe showed decreased fALFF, which was mainly related to cognitive function, such as visuospatial function, reading, memory, speech comprehension and executive function (Andrews-Hanna et al., 2010; Zhan et al., 2018). When these brain regions are impaired, cognitive dysfunction occurs (Mattila et al., 2000). Thus, it is possible that lower VD levels lead to greater brain damage in PD patients, resulting in more spontaneous activity enhancement in the affected brain cortex areas (DMN and visual pathway neurons) as compensatory responses. Furthermore, the decrease of spontaneous activity in a part of the temporal lobe may be compensated by increased spontaneous activity in other brain regions associated with cognitive function, such as the DMN. In this study, there were significant differences in cognitive scales (MMSE) between the PD + NVD and PD + VDD groups (Figure 2B). And comparison tests with Bonferroni correction found the differences in the years of education fell short of significance (Figure 2A). It may be due to serum VD may play an important role in maintaining cognitive ability, which is consistent with other published studies (Balion et al., 2012; Groves et al., 2014; Ouma et al., 2018). Previous studies have also shown that DMN dysfunction is a common pathological change in PD patients (Luo et al., 2014), which may also play a role in the progression of cognitive impairment in PD (Tessitore et al., 2012; Lucas-Jimenez et al., 2016). We couldn’t agree more that declined cognition itself could affect DMN pathway. But in this study, when compared with HC group, we found that there was a progressive trend in the changes of DMN spontaneous neuronal activity: PD + VDD group had the largest change, followed by PD + VDI group, and finally PD + NVD group. While it showed no such trend in the MMSE scores among the three groups (Figure 2B). Thus, the change of DMN cannot entirely be explained by the declined cognition. Together our results suggest that low levels of VD are associated with the DMN pathway, which may in turn impact on the cognition. However, these results still do not allow us to draw definite conclusions about relationship between VD and cognition in PD, and further validation studies should be conducted.

## Conclusion

Our study confirmed an inverse association between PD and 25(OH)D levels. Furthermore, we identified altered neural network function compared with control individuals, which was correlated with VD deficiency in PD patients, along with BOLD signal change in DMN and visual pathway neurons. These results confirm prior associations and provide additional evidence for a causal relationship between lower VD levels and PD, while the fMRI results add to the knowledge of the neural basis for the role of VD in PD.

## Limitations

This study has some limitations. First, the definition of optimal vitamin D status in central nervous system is elusive. In our study, the cut-off value of serum 25(OH)D level was determined based on bone health. In addition, this is a cross-sectional study that lacks longitudinal clinical observation of VD levels in PD patients, so it is not possible to obtain conclusive results on the relationship between VD and PD. Thus, more prospective and longitudinal cohort studies spanning different periods of PD are necessary to investigate if adequate VD intake can prevent or delay the onset of PD. Second, this was a small-sized rs-fMRI study after subgrouping, which limits inferences on any causal relationships between VD and PD risk. However, the resting-state fMRI measurements are highly consistent with the clinical results obtained in this study. Third, although the clinical evaluation and MRI scans of PD patients were performed in the “OFF” state, the influence of long-acting dopaminergic drugs on the results was unavoidable.

## Data Availability Statement

The raw data supporting the conclusions of this article will be made available by the authors, without undue reservation, to any qualified researcher.

## Ethics Statement

The studies involving human participants were reviewed and approved by the Ethics Committee of The Second Xiangya Hospital of Central South University. The patients/participants provided their written informed consent to participate in this study.

## Author Contributions

LL and HZ designed the study, analyzed the data, and wrote the manuscript. XT analyzed the data. LL, XP, RB, and QX were responsible for data collection and inputted and sorting during the whole process. CT, HL, WY, LQ, and BT discussed and revised the manuscript. CW provided guidance and suggestions during the entire research process. All authors contributed to the article and approved the submitted version.

## Conflict of Interest

The authors declare that the research was conducted in the absence of any commercial or financial relationships that could be construed as a potential conflict of interest.

## Publisher’s Note

All claims expressed in this article are solely those of the authors and do not necessarily represent those of their affiliated organizations, or those of the publisher, the editors and the reviewers. Any product that may be evaluated in this article, or claim that may be made by its manufacturer, is not guaranteed or endorsed by the publisher.

## Footnotes

1
http://rfmri.org/dpabi

## Abbreviations

OR: odds ratios
BOLD: blood oxygen level-dependent
DMN: default-mode network
fALFF: fraction amplitude of low-frequency fluctuation
HC: healthy controls
H&Y: Hoehn and Yahr Staging Scale
LEDD: levodopa equivalent daily dose
MMSE: Mini-Mental State Examination
PD: Parkinson’s disease
PD + VDD: Parkinson’s disease with vitamin D deficiency
PD + VDI: Parkinson’s disease with vitamin D deficiency
PD + NVD: Parkinson’s disease with normal vitamin D
Rs-fMRI: resting-state functional magnetic resonance imaging
UPDRS-III: unified Parkinson’s disease Rating Scale part III
VD: vitamin D.
